# A retrospective case-control study of hepatitis C virus infection and oral lichen planus in Japan: association study with mutations in the core and NS5A region of hepatitis C virus

**DOI:** 10.1186/1471-230X-12-31

**Published:** 2012-04-10

**Authors:** Yumiko Nagao, Michio Sata

**Affiliations:** 1 Department of Digestive Disease Information & Research, Kurume University School of Medicine, Kurume, Fukuoka 830-0011, Japan; 2 Division of Gastroenterology, Department of Medicine, Kurume University School of Medicine, Kurume, Fukuoka 830-0011, Japan

## Abstract

**Background:**

The aims of this study were to assess the prevalence of hepatitis C virus (HCV) infection in Japanese patients with oral lichen planus and identify the impact of amino acid (aa) substitutions in the HCV core region and IFN-sensitivity-determining region (ISDR) of nonstructural protein 5A (NS5A) associated with lichen planus.

**Methods:**

In this retrospective study, 59 patients (group 1-A) with oral lichen planus among 226 consecutive patients who visited our hospital and 85 individuals (group 1-B, controls) with normal oral mucosa were investigated for the presence of liver disease and HCV infection. Risk factors for the presence of oral lichen planus were assessed by logistic regression analysis. We compared aa substitutions in the HCV core region (70 and/or 91) and ISDR of NS5A of 12 patients with oral lichen planus (group 2-A) and 7 patients who did not have oral lichen planus (group 2-B) among patients (high viral loads, genotype 1b) who received interferon (IFN) therapy in group1-A.

**Results:**

The prevalence of anti-HCV and HCV RNA was 67.80% (40/59) and 59.32% (35/59), respectively, in group 1-A and 31.76% (27/85) and 16.47% (14/85), respectively, in group 1-B. The prevalence of anti-HCV (*P *< 0.0001) and HCV RNA (*P *< 0.0001) in group 1-A was significantly higher than those in group 1-B. According to multivariate analysis, three factors - positivity for HCV RNA, low albumin level (< 4.0 g/dL), and history of smoking - were associated with the development of oral lichen planus. The adjusted odds ratios for these three factors were 6.58, 3.53 and 2.58, respectively, and each was statistically significant. No significant differences in viral factors, such as aa substitutions in the core region and ISDR of NS5A, were detected between the two groups (groups 2-A and -B).

**Conclusion:**

We observed a high prevalence of HCV infection in patients with oral lichen planus. Longstanding HCV infection, hypoalbuminemia, and smoking were significant risk factors for the presence of oral lichen planus in patients. It is advisable for Japanese patients with lichen planus to be tested for HCV infection during medical examination.

## Background

Hepatitis C virus (HCV) infection is a major public health problem because it causes chronic hepatitis, cirrhosis and hepatocellular carcinoma (HCC). In Japan, elderly patients are at a higher risk for HCC and hepatitis C viral eradication has a smaller effect on hepatocarcinogenesis in older patients [1]. In Japan, refractory cases with genotype 1b and high viral loads are seen in as many as 70% of chronic hepatitis C patients.

Recent studies have identified both viral and host factors predictive of the outcome of interferon (IFN) therapy. Among the viral factors, the number of substitutions in the region amino acids (aa) 2209-2248 (IFN-sensitivity-determining region, ISDR, of nonstructural protein 5A, NS5A, in HCV genotype 1b) is associated with a sustained virological response (SVR) after IFN treatment of HCV genotype 1b patients [2,3]. In addition, Akuta et al. reported that aa substitution at position 70 and/or 91 in the HCV core region in patients infected with HCV genotype 1b are independent predictors of SVR and non-virological response (NVR) [4,5] and also affect hepatocarcinogenesis [6].

HCV mainly affects the liver but there are several extrahepatic manifestations of chronic HCV infection, including hematologic diseases such as cryoglobulinemia and lymphoma, renal disease such as membranoproliferative glomerulonephritis, autoimmune disorders such as thyroiditis, and dermatologic conditions such as lichen planus and porphyria cutanea tarda [7-10].

Lichen planus is a chronic inflammatory disease that affects the skin and oral mucosa. The association of lichen planus with HCV infection has been reported widely in the literature [11-16]. In particular, the relationship between lichen planus and HCV has been suggested by studies from Japan, Italy, Spain, and Brazil, indicating a strong geographic relationship [11-16]. We first reported a relationship between HCV and lichen planus in Japan [12]. Previous reports indicated that there are no differences in the viral factors such as viral load and genotype between HCV-infected patients with lichen planus and those without [17,18].

This study was conducted to determine the rate of HCV infection in individuals with lichen planus, and with normal oral mucosa as a control group, and to investigate the impact of aa substitutions in the HCV core region (70 and/or 91) and ISDR of NS5A among HCV-infected patients with lichen planus and those without.

## Materials and methods

### Group 1 (Patients)

A total of 226 consecutive patients had checkups for oral mucosal diseases in the Digestive Diseases Center at Kurume University Hospital Japan from February 2, 2010 to June 28, 2011. In this Digestive Diseases Center, physicians, surgeons, radiologists and an oral surgeon examine each patient in their own specialized area. An oral surgery specialist diagnosed 59 of 226 patients with oral lichen planus clinically and/or histopathologically. These 59 patients then consulted a liver specialist in the same center for the presence or absence of liver disease. The 59 patients (group 1-A) ranged in age from 32 to 87 years, with an average age of 68.05 ± 9.93 years. There were 14 men and 45 women.

### Group 1 (Controls)

We have continued carrying out health screenings of the residents of X town (adult population: 7,389), in northern Kyushu, Japan where the prevalence of HCV infection is the highest in the country [19]. We reported previously that the residents had a high prevalence of HCV infection, that HCV infection was the principal cause of liver disorders, and that extrahepatic manifestations were strongly associated with HCV infection [19-28].

In the previous study of 509 residents selected randomly, the prevalence of antibodies to HCV (anti-HCV), HCV RNA, and hepatitis B surface antigen (HBsAg) was 23.6%, 17.9%, and 2.6%, respectively [19]. We conducted cohort studies from August 1990 to May 2002 on the long-term prognosis of HCV carriers based on the same subjects in the town and investigated the risk factors for deaths due to HCC and liver cirrhosis [24,25].

One hundred and thirty nine subjects who had received a liver disease examination in 2002 were interviewed in person by two trained interviewers and all but one of them were given an oral mucosal examination by an oral surgeon. We used the headband fiber (50-100-10, Daiichi Medical Co., Ltd.) that had a brightness of 34,000 luces for mucosal examination. In this report, we used a control group consisting of 85 age- and gender- matched individuals (group 1-B) with normal oral mucosa, selected from the 139 subjects. The individuals ranged in age from 52 to 90 years, with an average age of 66.35 ± 9.83 years. We inquired about the following information: present health condition, medical treatment received in the hospital, name of the family doctor, the kind of medicine taken, and the presence of extrahepatic manifestations of HCV infection such as diabetes mellitus and thyroid dysfunction. We also asked the family doctors of the subjects about the presence of extrahepatic manifestations.

### Group 2 (Patients)

The subjects were 19 patients with high titers of genotype 1b (over 100 KIU/mL or 5.0 log IU/mL) who were investigated for aa substitutions in the HCV core region (70 and/or 91) and ISDR of NS5A. These were non-SVR patients among the 226 patients of group 1 who were not able to eradicate an HCV during past IFN therapy. We compared the characteristics of 12 patients who had lichen planus (group 2-A) and 7 patients who did not have lichen planus (group 2-B). Group 2 comprised 5 men and 14 women ranging in age from 44 to 73 years (average 64.47 ± 8.48).

## Methods

### Serological assays

Serum samples from the 144 subjects, including 59 patients with lichen planus and 85 people with normal oral mucosa, were tested for platelets (PLT) and hemoglobin (Hb), and for the following liver function tests: serum alanine aminotransferase (ALT), aspartate aminotransferase (AST), lactate dehydrogenase (LDH), alkaline phosphatase (ALP), total bilirubin (T.Bil), total cholesterol (TC), total protein (TP), and albumin (Alb).

### Evaluation of liver diseases

Anti-HCV was measured using a chemiluminescent enzyme immunoassay kit (Lumipulse II HCV, Fujirebio, Tokyo, Japan). HCV RNA in serum was analyzed by the Amplicor HCV test (Roche, Tokyo, Japan) up to December 6, 2007 and by quantitative PCR assay (COBAS AMPLICOR HCV MONITOR v 2.0 Test, COBAS AmpliPrep/COBAS Taq-Man HCV Test, Roche Molecular Systems, New Jersey, US) from December 7, 2007 [29,30]. HCV genotype was determined by polymerase chain reaction assay, using a mixture of primers for the subtype, as reported previously [31]. HBsAg was assayed using a chemiluminescent immunoassay kit (Architect, HBsAg QT, Dainabot, Tokyo, Japan). Ultrasonographic examination was performed on 59 patients and 27 control inhabitants infected with HCV in order to investigate the shape of the liver and lesions occupying the liver. Computed tomography and liver biopsy were performed on some patients. We used other possible predictors of liver cirrhosis progression, including serum Alb, T.Bil, prothrombin time, and PLT.

### Measurement of body weight

Obesity was defined as a body mass index (BMI) > 25 kg/m^2^

### Detection of amino acid substitutions in the core and NS5A regions

Detection of aa substitutions in the HCV core region was performed using the method reported previously [4,5] and the patients were divided into two groups on the basis of the findings. Patients with substitution of aa 70 in the core region of HCV were assigned to the mutant type group and patients without this substitution were assigned to the wild type group. The ISDR of NS5A was analyzed using the method reported previously [3]. Strains with no aa changes in the ISDR, with 1 to 3 aa changes, and with > 3 aa changes were designated as wild type, intermediate type, and mutant type, respectively.

### Ethics considerations

The study protocol was approved by the Ethics Committee of Kurume University (reference number: 2134-1) in accordance with the Declaration of Helsinki. Written informed consent for participation in the study was obtained from each patient.

### Statistical analysis

All data are expressed as mean ± standard error. Differences between the two groups were analyzed using the Mann-Whitney U test, Wilcoxon's test, and the Fisher's exact test. Differences were judged significant for *p *< 0.05 (two-tailed). Adjusted odds ratios were calculated using logistic regression analysis. All statistical analyses were conducted using JMP Version 6 (SAS Institute, Cary, NC, USA). The level of statistical significance was defined as 0.05.

## Results

### Prevalence of HCV infection among patients with lichen planus: a retrospective case-control study

We compared the characteristics of group 1-A and group 1-B. The characteristics of the 144 subjects studied are shown in Table 1. The prevalence of anti-HCV and HCV RNA was 67.80% (40/59) and 59.32% (35/59), respectively, in group 1-A and 31.76% (27/85) and 16.47% (14/85), respectively, in group 1-B. The prevalence of anti-HCV (*P *< 0.0001) and HCV RNA (*P *< 0.0001) in group 1-A was significantly higher than those in group 1-B.

**Table 1:** Characteristics of study population 1 (n = 144)

Characteristics		Total		Group1-A		Group1-B		A vs. B
				**with lichen planus**		**with normal oral mucosa**		**P value**

No. subjects		144		59		85		

Sex	male/female	39/105		14/45		25/60		NS

Age	(mean ± SD), years	67.05 ± 9.87		68.05 ± 9.93		66.35 ± 9.83		NS

Liver disease	positive (%)	73	50.69%	46	77.97%	27	31.76%	< 0.0001

Diagnosis of liver diseases	Past history of HCV infection	13	9.03%	1	1.69%	12	14.12%	

	CH-C	31	21.53%	20	33.90%	11	12.94%	

	CH-C during IFN	4	2.78%	4	6.78%	0	0.00%	

	CH-C post IFN (SVR)	5	3.47%	4	6.78%	1	1.18%	

	CH-C + AIH	1	0.69%	1	1.69%	0	0.00%	

	CH-C + post HCV-related HCC	3	2.08%	2	3.39%	1	1.18%	

	LC-C	3	2.08%	2	3.39%	1	1.18%	

	LC-C + post HCV-related HCC	7	4.86%	6	10.17%	1	1.18%	

	CH-B	1	0.69%	1	1.69%	0	0.00%	

	LC-B	1	0.69%	1	1.69%	0	0.00%	

	PBC	1	0.69%	1	1.69%	0	0.00%	

	Fatty liver	2	1.39%	2	3.39%	0	0.00%	

	Fatty liver + ALD	1	0.69%	1	1.69%	0	0.00%	

	Normal liver	71	49.31%	13	22.03%	58	68.24%	

Diabetes mellitus	positive (%)	14	9.72%	6	10.17%	8	9.41%	NS

Thyroid disease	positive (%)	8	5.56%	6	10.17%	2	2.35%	0.0440

Extrahepatic malignant tumor	positive (%)	15	10.42%	5	8.47%	10	11.76%	NS

Smoking history	yes (%)	37	25.69%	22	37.29%	15	17.65%	0.0080

History of alcohol intake	yes (%)	59	40.97%	19	32.20%	40	47.06%	NS

Anti-HCV	positive (%)	67	46.53%	40	67.80%	27	31.76%	< 0.0001

HCV RNA	positive (%)	49	34.03%	35	59.32%	14	16.47%	< 0.0001

HCV RNA level	High	46	85.19%	32	82.05%	14	93.33%	NS

	Low	3	5.56%	3	7.69%	0	0.00%	NS

	unexmained	5	9.26%	4	10.26%	1	6.67%	

HCV genotype	1b	27	55.10%	27	77.14%	-		

	2a	2	4.08%	2	5.71%	-		

	2b	0	0.00%	0	0.00%	-		

	unexmained	20	40.82%	6	17.14%	14	100.00%	

HBsAg	positive (%)	3	2.08%	2	3.39%	1	1.18%	NS

BMI		22.70 ± 3.20		22.44 ± 3.20		22.88 ± 3.20		NS

AST	(U/L) (mean ± SD)	29.71 ± 16.86		36.09 ± 20.33		25.65 ± 12.78		0.0009

ALT	(U/L) (mean ± SD)	25.47 ± 20.06		30.54 ± 22.39		22.25 ± 17.82		0.0031

LDH	(U/L) (mean ± SD)	202.40 ± 43.56		211.42 ± 54.63		196.88 ± 34.34		NS

ALP	(U/L) (mean ± SD)	272.76 ± 112.57		299.62 ± 155.99		256.33 ± 70.88		NS

TP	(g/dL) (mean ± SD)	7.17 ± 0.42		7.27 ± 0.43		7.11 ± 0.41		NS

Alb	(g/dL) (mean ± SD)	4.15 ± 0.33		3.99 ± 0.38		4.26 ± 0.25		< 0.0001

T.Bil	(mg/dL) (mean ± SD)	1.13 ± 5.27		2.03 ± 8.57		0.60 ± 0.20		< 0.0001

TC	(mg/dL) (mean ± SD)	192.34 ± 35.28		179.12 ± 37.18		200.12 ± 31.84		0.0007

Hb	(g/dL) (mean ± SD)	13.30 ± 1.39		13.16 ± 1.39		13.39 ± 1.38		NS

PLT	(/μL) (mean ± SD)	18.30 ± 5.57		15.95 ± 6.42		19.71 ± 4.48		< 0.0001

*HCV*, hepatitis C virus; *CH-C*, chronic hepatitis C; *LC-C*, liver cirrhosis type C; *HCC*, hepatocellular carcinoma; *CH-B*, chronic hepatitis B; *LC-B*, liver cirrhosis type B; *AIH*, autoimmune hepatitis; *PBC*, primary biliary cirrhosis; *ALD*, alcoholic liver disease; *IFN*, interferon; *NS*, not significant; *AST*, aspartate aminotransferase; *ALT*, alanine aminotransferase; *LDH*, lactate dehydrogenase; *ALP*, alkaline phosphatase; *TP*, total protein; *Alb*, albumin; *T.Bil*, total bilirubin; *TC*, total cholesterol; *PLT*, platelets

The prevalence of liver disease (*P *< 0.0001), thyroid disease (*P *= 0.0440) and history of smoking (*P *= 0.0080) was significantly higher in group 1-A than in group 1-B (Table 1).

The diagnosis of liver disease in group 1-A included: past history of HCV infection (n = 1), chronic hepatitis C (n = 20), chronic hepatitis C during IFN therapy (n = 4), SVR after IFN therapy (n = 4), chronic hepatitis C with autoimmune hepatitis (n = 1), chronic hepatitis C with post-treatment of HCC (n = 2), HCV-related liver cirrhosis (n = 2), HCV-related liver cirrhosis with post-treatment of HCC (n = 6), chronic hepatitis B (n = 1), HBV-related liver cirrhosis B (n = 1), primary biliary cirrhosis (PBC) (n = 1), fatty liver (n = 2), and fatty liver with alcoholic liver disease (n = 1). Those of group 1-B included: past history of HCV infection (n = 12), chronic hepatitis C (n = 11), SVR after IFN therapy (n = 1), chronic hepatitis C with post-treatment of HCC (n = 1), HCV-related liver cirrhosis (n = 1), and HCV-related liver cirrhosis with post-treatment of HCC (n = 1).

We analyzed the differences between these two groups in terms of laboratory values for liver function tests and PLT. As shown in Table 1, abnormal levels of AST (*P *= 0.0009), ALT (*P *= 0.0031), Alb (*P *< 0.0001), T.Bil (*P *< 0.0001), TC (*P *= 0.0007), and PLT (*P *< 0.0001) differed significantly between the groups.

We examined the viral load in 39 of 40 anti-HCV-positive patients, excluding one patient who cleared serum HCV RNA naturally (group 1-A). In seven patients, including during IFN therapy (4 patients) and SVR (3 of 4), the viral load was measured before IFN therapy. The HCV RNA level was high viral load (over 100 KIU/mL or 5.0 log IU/mL) in 32 (82.05%) group 1-A patients, low viral load (less than 100 KIU/mL or 5.0 log IU/mL) in 3 (7.69%) and unknown in 4 (10.26%). All HCV RNA-positive individuals among 14 members of group 1-B had a high viral load. As for an SVR case in the group 1-B, pretreatment viral load was not clear. We also examined the HCV genotype of group 1-A; the genotype was 1b in 27 (77.14%) patients, 2a in two (5.71%), and unknown in 6 (17.14%). The genotype of group 1-B was not measured.

### Multivariate analysis

In multivariable analysis, all variables in the univariable analyses were included. According to multivariate analysis, three factors, positivity for HCV RNA, low albumin level (< 4.0 g/dL), and history of smoking, were associated with the development of lichen planus. The adjusted odds ratios for these three factors were 6.58, 3.53 and 2.58, respectively, and each was statistically significant (Table 2).

**Table 2:** Factors associated with lichen planus according to multivariate analysis

	Adjusted odds ratio	(95% confidence interval)	P value
HCV RNA positive	6.58	(2.89 - 15.69)	< 0.0001
Albumin < 4.0 g/dL	3.53	(1.42 - 9.05)	0.0072
History of smoking (yes)	2.58	(1.05 - 6.48)	0.0396

### Detection amino acid substitutions in the HCV core region and ISDR of NS5A among HCV-infected patients with lichen planus and those without

In the present study, aa substitutions in the core region (70 and/or 91) and ISRR of NS5A were analyzed by direct sequencing in 19 HCV-infected patients with lichen planus and those without [3-5] (Table 3). No significant differences were seen between the two groups (groups 2-A and -B).

**Table 3:** Characteristics of study population 2 (n = 19)

Characteristics			Group2-A with lichen planus (n = 12)	Group2-AB without lichen planus (n = 7)	P value
Age (mean ± SD), years			64.83 ± 7.51	63.86 ± 10.57	NS
Sex (male/female)			4/8	1/6	NS
Diagnosis of liver diseases	CH-C (CH-C post HCC)		10 (2), 83.33%	7 (100%)	NS
	LC-C (LC-C post HCC)		2 (1), 16.67%	0 (0%)	NS
Viral factors	HCV RNA level (logIU/mL)	High	12 (100%)	7 (100%)	NS
	HCV genotype	1b	12 (100%)	7 (100%)	NS
	Amino acid substitutions in the core region	aa 70 (wild type/non-wild)	5/7 (41.67%/58.33%)	2/5 (28.57%/71.43%)	NS
		aa 91 (wild type/non-wild)	6/6 (50.00%/50.00%)	5/2 (71.43%/28.57%)	NS
		aa 70 and 91(double-wild type/non-double-wild type)	3/9 (25.00%/75.00%)	1/6 (14.29%/85.71%)	NS
	Number of ISDR substituions	0 (wild type)	8 (66.67%)	3 (42.86%)	NS
		1-3 (intermediate type)	4 (33.33%)	4 (57.14%)	NS
Effect of previous IFN therapy (non-SVR)			12 (100%)	7 (100%)	NS

*HCV*, hepatitis C virus; *CH-C*, chronic hepatitis C; *LC-C*, liver cirrhosis type C; *HCC*, hepatocellular carcinoma; *IFN*, interferon; *SVR*, sustained virological response; *ISDR, IFN *sensitivity-determining region; *NS*, not significant

## Discussion

In this study, we demonstrated that HCV infection is significantly associated with the development of lichen planus. The odds ratio was 6.58 (95% confidence interval 2.89-15.69). This odds ratio was higher than that of a meta-analysis published recently (odds ratio 2.8) [32]. Hypoalbuminemia and smoking also increased the risk of lichen planus. However, substitutions of aa 70 and/or 91 and ISDR regions had no impact on the development of lichen planus.

HCC is one of the most common malignancies, especially in Japan. The most important risk factors of HCC are chronic hepatitis C, B and cirrhosis. The effect of Peg-IFN and ribavirin (RBV) treatment on chronic HCV infection has been established in several controlled clinical studies [33]. The effects of IFN are summarized as follows: elimination of HCV, reduction of hepatic inflammation, improvement in liver fibrosis, and reduction in hepatocarcinogenesis [34]. In Japan, 70% of refractory cases are infected with genotype 1b with a high HCV RNA load. Japanese patients with chronic HCV infection are getting older. Viral mutations leading to aa substitutions in the HCV core region (70 and/or 91) and ISDR of NS5A have been shown to be valuable as predictors of virological response to combination therapy with Peg-IFN and RBV [2-6]. In addition, single nucleotide polymorphisms (SNPs) around the human interleukin 28B (IL28B) gene locus have been reported to affect the efficacy of Peg-IFN and RBV therapy in chronic hepatitis with HCV genotype 1b infection [35,36]. In Japan, there is an established standard therapy for chronic hepatitis C based on guidelines including viral factors such as genotype and levels of HCV RNA. More recently, the effects of variation of IL28B and aa substitutions in the HCV core region and ISDR of NS5A have been established.

The association between lichen planus and HCV infection seems to be more common in individuals living in areas of Japan and Italy [11-13]. The difference in the prevalence HCV-infection in patients with lichen planus seems to be due to geographic differences in HCV infection, selection of study subjects such as sex, average age, and diagnosis in the respective countries, and some genetic predisposition such as the HLA-DR6 allele. The HLA-DR6 allele is reported to be significantly more common in Italian patients with lichen planus and HCV [37]. Moreover, not all reported studies included screening of hepatic damage and measurement of serum HCV RNA to determine the presence or absence of hepatic diseases. The areas of our study are in the northern Kyushu region, where the HCV infection rate and the mortality rate from HCC are the highest in Japan. Previous reports indicated that there are no differences in the viral factors such as viral load and genotype between HCV-infected patients with lichen planus and those without [17,18]. This study ruled out a direct association of viral factors, such as substitutions of aa 70 and/or 91 and in the ISDR region, with the development of HCV-related lichen planus.

We reported that the prevalence of HCV-related lichen planus increased as the subjects grew older in Japan [38]. We also reported previously the prevalence of HCV infection among lichen planus among 45 patients who visited the Department of Oral Surgery at our hospital from November 1993 to April 1994 [12]. The mean age (68.1 years) of the 59 patients (group 1-A) in the present study was higher than that (61.1 years) of the previous 45 patients.

We demonstrated a strong association between hypoalbuminemia and mortality in a hyperendemic area (X town) of HCV infection in Japan [39]. Residents with hypoalbuminemia had a mortality of 68.0%; dramatically higher than the rate of 12.1% among residents who had normal albumin levels. Hypoalbuminemia was an independent risk factor for the development of lichen planus. Recent studies have demonstrated the efficacy of branched-chain amino acid (BCAA) supplementation in improving hypoalbuminemia in cirrhotic patients [40]. The effects of BCAA intake have been investigated in a number of disease models, including obesity and metabolic disorders, liver disease, impaired immunity, muscle atrophy, cancer, and a variety of injury (postoperative, trauma, burn, and sepsis) [41]. Among Japan's aging population, BCAA intake seems to be useful for HCV-infected patients with lichen planus.

## Conclusion

In conclusion, our data show that HCV infection could be the main pathogenic factor of lichen planus in Japanese patients. Routine HCV testing and medical examination for lichen planus are recommended for patients in high-risk HCV areas like Japan. Further investigation is required to clarify the mechanisms of development of HCV-related lichen planus.

## Abbreviations

HCV: Hepatitis C virus; CH-C: Chronic hepatitis C; CH-B: Chronic hepatitis B; LC-C: Liver cirrhosis type C; LC-B: Liver cirrhosis type B; HCC: Hepatocellular carcinoma; PBC: Primary biliary cirrhosis; IFN: Interferon; RBV: Ribavirin; SVR: Sustained virological response; NVR: Non-virological response; aa: Amino acids; ISDR: IFN-sensitivity-determining region; NS5A: Nonstructural protein 5A; PLT: Platelets; Hb: Hemoglobin; ALT: Alanine aminotransferase; AST: Aspartate aminotransferase; LDH: Lactate dehydrogenase; ALP: Alkaline phosphatase; T.Bil: Total bilirubin; TC: Total cholesterol; TP: Total protein; Alb: Albumin

## Competing interests

The authors declare that they have no competing interests.

## Authors' contributions

YN carried out most of the data collection and drafted the manuscript. MS contributed to data analysis. All authors read and approved the final manuscript.

## Source of support

This study was supported in part by a Grant-in-Aid for Scientific Research (C) (No.22592354) from the Ministry of Education, Culture, Sports, Science and Technology of Japan.

## Pre-publication history

The pre-publication history for this paper can be accessed here:

http://www.biomedcentral.com/1471-230X/12/31/prepub
